# Editorial: Reviews in takotsubo syndrome

**DOI:** 10.3389/fcvm.2024.1446689

**Published:** 2024-07-01

**Authors:** Tomohiro Hayashi

**Affiliations:** ^1^ Division of Community Medicine and Career Development, Kobe University Graduate School of Medicine, Kobe, Japan; ^2^ Department of Internal Medicine, Hyogo Prefectural Tamba Medical Center, Tamba, Japan

**Keywords:** takotsubo syndrome, pathophysiology, cancer, animal model, treatment

**Editorial on the Research Topic** Reviews in takotsubo syndrome

Takotsubo syndrome (TTS) is an acute cardiac condition characterized by transient left ventricular systolic dysfunction and regional wall motion abnormalities in the absence of obstructive coronary disease or angiographic evidence of acute plaque rupture. It was first conceptualized by Dr. Sato in 1990 (1). Previously thought to be a benign condition, TTS is now recognized to be associated with a substantial in-hospital mortality rate (2), wherein the mortality risk in long-term patients with TTS is equivalent to that of acute myocardial infarction, with an all-cause mortality rate of 5.6% per patient-year (3–5). Moreover, the recurrence rate of TTS has been reported to be 4%–5% (6, 7). Despite several mechanisms being identified in clinical and basic research (i.e., endogenous adrenergic surge, brain-heart axis, coronary vasospasm and microvascular reactivity, metabolic and energetic alterations, and inflammation) (8–11) to explain the syndrome, and some clinical trials assessing potential interventions for TTS are underway, there are no evidence-based treatments for TTS to date. In four reviews, the current research topic of “Reviews in takotsubo syndrome” nicely summarized important clinical and translational knowledge regarding TTS, thereby contributing to our understanding of its pathogenesis and pathophysiology.

In the first review, Osawa et al. conducted a systemic review and meta-analysis of 14 studies (189,210 patients) published in PubMed and Cochrane Library databases up to December 2022 to investigate the outcomes of patients with TTS and cancer. The authors demonstrated that 8.7% of patients with TTS had current or previous malignancy. Patients with TTS and malignancy had a higher risk of all-cause mortality at the longest follow-up than those with TTS alone. Cancer was significantly associated with an increased risk of in-hospital or 30-day mortality, in-hospital shock, the need for mechanical respiratory support, arrhythmia, and major adverse cardiac events. In contrast, the need for mechanical circulatory support and the risk of cardiovascular death did not differ between TTS patients with and without cancer. In another review, Tini et al. also provided an overview of contemporary knowledge on the association between TTS and cancer. Although the definition of cancer varies among studies, its prevalence in patients with TTS ranges from 7% to 35%. Several studies have demonstrated an association between cancer and all-cause mortality in patients with TTS. Overall, these two review papers highlight the significant associations between TTS and cancer, underscoring the importance of close collaboration between cardiologists and oncologists to care for patients with TTS and cancer.

Midventricular TTS (MV-TTS), characterized by wall motion abnormalities of the middle portion of the left ventricle along with normokinesia or hyperkinesia of the apical and basal portions, is the most frequent atypical subtype. Padilla-Lopez et al. examined electrocardiograms (ECGs) at three time points (within the first 12 h, at 48 h, and at 5–7 days from symptom onset) in patients with typical TTS (*n* = 33) and those with MV-TTS (*n* = 27), as classified by ventriculography. Patients with MV-TTS exhibited a distinctive pattern of ECG abnormalities compared to typical TTS: (1) ST-segment depression in inferolateral leads in the acute phase; (2) less profound and less extensive T-wave inversion that mostly affected leads I, aVL, and V2; and (3) attenuated QT interval prolongation.

In the present issue, Zulfaj et al. reviewed and comprehensively evaluated current animal models of TTS, focusing on their ability to replicate key clinical trials and identifying limitations. In addition, the authors summarized clinical characteristics and potential mechanisms of TTS and introduced a promising novel animal model of TTS. Collectively, this review provides a guide for researchers using animal models of TTS, enhancing translational validity.

In summary, this research topic highlights important clinical features of TTS and provides further insights to help us understand its mechanisms in clinical practice. The topic also emphasizes the need for mechanistic translational studies using appropriate animal models, aimed at unveiling precise underlying pathophysiology and developing evidence-based treatment options specifically for TTS in order to improve the prognosis of this syndrome.
